# Interaction between *Calpain 5, Peroxisome proliferator-activated receptor-gamma *and *Peroxisome proliferator-activated receptor-delta *genes: a polygenic approach to obesity

**DOI:** 10.1186/1475-2840-7-23

**Published:** 2008-07-25

**Authors:** María E Sáez, Antonio Grilo, Francisco J Morón, Luis Manzano, María T Martínez-Larrad, Antonio González-Pérez, Javier Serrano-Hernando, Agustín Ruiz, Reposo Ramírez-Lorca, Manuel Serrano-Ríos

**Affiliations:** 1 Departamento de Genómica Estructural, Neocodex, C/.,Charles Darwin 6, Acc. A, 41092, Sevilla, Spain; 2 Departamento de Medicina Interna, Unidad de Hipertensión, Hospital Universitario de Valme, Ctra, Sevilla-Cádiz S/N, 41014, Sevilla, Spain; 3 Departamento de Medicina Interna, Unidad de Insuficiencia Cardiaca y Riesgo Vascular, Hospital Ramón y Cajal, Crta, De Colmenar Viejo Km, 9100, 28034, Madrid, Spain; 4 Departamento de Angiología Vascular, Hospital Clínico San Carlos, C/Profesor Martín Lagos s/n, 28040, Madrid, Spain; 5 Departamento de Medicina Interna II, Hospital Clínico San Carlos, C/Profesor Martín Lagos s/n, 28040, Madrid, Spain

## Abstract

**Context:**

Obesity is a multifactorial disorder, that is, a disease determined by the combined effect of genes and environment. In this context, polygenic approaches are needed.

**Objective:**

To investigate the possibility of the existence of a crosstalk between the *CALPAIN 10 *homologue *CALPAIN 5 *and nuclear receptors of the peroxisome proliferator-activated receptors family.

**Design:**

Cross-sectional, genetic association study and gene-gene interaction analysis.

**Subjects:**

The study sample comprise 1953 individuals, 725 obese (defined as body mass index ≥ 30) and 1228 non obese subjects.

**Results:**

In the monogenic analysis, only the peroxisome proliferator-activated receptor delta (*PPARD*) gene was associated with obesity (OR = 1.43 [1.04–1.97], p = 0.027). In addition, we have found a significant interaction between *CAPN5 *and *PPARD *genes (p = 0.038) that reduces the risk for obesity in a 55%.

**Conclusion:**

Our results suggest that *CAPN5 *and *PPARD *gene products may also interact in vivo.

## Introduction

Obesity, which is among of the most hereditable human conditions (45–75%), is, with few exceptions, a polygenic disorder determined by an unknown number of genes with mild to moderate individual effects that also interacts with the environment to produce the phenotype. The importance of the genetic component in obesity has been assessed by the observation of familial aggregation, prevalence differences among ethnic groups and concordance rates in twin studies [1]. Association analysis has been the more successful strategy for the identification of these genetic factors, but these studies are not always replicated in subsequent analyses and only a small proportion of these genes have been consistently associated with the disease [2]. This situation is mainly due to small single gene effects, reason why, genetic association analysis can be strongly influenced by sample size, population structure and allele frequency. Thus, the polygenic approach is arising as the next landmark for the study of common complex diseases [3,4].

The peroxisome proliferator-activated receptors (PPARs) belong to a nuclear receptor (NR) superfamily of ligand-inducible transcription factors that form functional complexes with the retinoid × receptors (RXRs). Three subtypes, alpha (α), gamma (γ) and delta (δ), have been identified. PPARα (PPARA) is a regulator of fatty acid oxidation [5], whereas PPARγ (PPARG) mainly functions as a regulator of adipogenesis [6]. *PPARG *is expressed at adipose tissue, macrophages and muscle where it regulates glucose metabolism. Knockout mice for *Pparg *are embryonic lethal, but specific deletion of the *PPARG *gene in fat and muscle has been shown to cause insulin resistance [7,8]. PPARG contributes to the control of energy expenditure by the induction of a futile metabolic cycle in the mitochondria mediated by glycerol kinase and the uncoupling proteins (UCPs), which are transcriptionally regulated by PPARG [9]. Genetic studies have also greatly contributed to corroborate the role of *PPARG *in the pathogenesis of metabolic syndrome related phenotypes: the Pro12Ala polymorphism of *PPARG *gene has been confirmed to be associated with greater body mass index (BMI) and insulin sensitivity in the obese subgroup in a recent meta-analysis with more than 32.000 individuals[10].

In the last few years, PPARδ (PPARD) has been shown to have a critical role in the regulation of energy metabolism [11]. Increased *Ppard *expression in adipose tissue in mice has a protective effect against elevated adiposity and serum lipid levels [12]. The activation of PPARD results in the increased expression of genes involved in lipid uptake, fatty acid oxidation and uncoupling proteins, highlighting the PPARD prominent role in mitochondrial activity [13,14]. *PPARD *gene variants have been associated with obesity [15-17], although some authors have failed to replicate this finding [18-21]. Recent reports have associated *PPARD *polymorphisms with the effectiveness of cardiovascular fitness, demonstrating the pivotal role of *PPARD *gene variants in mitochondrial function and, in consequence, in weight control [22,23].

The calpain family comprises a heterogeneous group of cysteine proteases with a broad expression pattern that includes multiple isoforms that are both ubiquitous and tissue specific. Calpains are involved in a variety of calcium-regulated cellular processes, such as signal transduction, cell proliferation and differentiation, and apoptosis [24]. The calpain role in apoptosis is related to the proteolytic cleavage of the proapoptotic Bax protein, a member of the Bcl-2 family, and the generation of a potent proapoptotic fragment (Bax/p18), which in turn mediates cytocrome c release and initiates the apoptotic execution; this action occurs in the mitochondria, so calpain activity exists within this cell fraction [25]. Calpain 10 (CAPN10) is a member of this protease family which is included into the atypical calpain subgroup, characterized by the absence of the EF-hand domain distinctive of the classical calpains and the presence of a so-called T domain homologue of the *Caenorhabditis elegans *TRA-3 calpain. The *CAPN10 *gene was identified as a type 2 diabetes (T2DM) susceptibility *locus *by Horikawa *et al*. [26] and, since then, has been associated with many other related conditions such as polycystic ovary syndrome (PCOS), dyslipidemia, hypertension or increased BMI by different groups [24]. CAPN10 has been related to pancreatic β-cell apoptosis initiated by the fatty acid palmitate [27]. Later on, CAPN10 protein was identified within the mitochondria: the overexpression of mitochondrial CAPN10 results in mitochondrial swelling and autophagy through the cleavage of Complex I subunits and activation of mitochondrial permeability transition (MPT) [28]. The *CAPN10 *homologue calpain 5 (*CAPN5*), was firstly analysed in humans by our group [29,30]. In these studies, we found that specific *CAPN5 *haplotypes could modify the risk of developing metabolic syndrome and PCOS, a common endocrine disorder in women of reproductive age in which all components of metabolic syndrome are frequent. *CAPN5 *haplotypes were also shown to be associated with diastolic blood pressure and cholesterol levels and a trend for BMI association was observed.

Interestingly, some reports have described proteolytic activity of calpains over the PPARs partners in signalling RXRs at the mitochondria [31,32] and over PPARG at the adipose tissue [33]. On the other hand, transcriptional regulation of calpain activity by PPARG has been also described [34].

Our main objective was to investigate the possibility of the existence of a crosstalk between the CAPN10 homologue CAPN5 and NRs of the PPAR family. With this purpose, we have selected highly informative polymorphisms in *CAPN5, PPARG *and *PPARD *genes and analysed it in 1953 individuals in relation to the absence or presence of obesity. Additionally, we have investigated the individual contributions of these polymorphisms to the obesity phenotype. We have found that the *PPARD *polymorphism is associated with obesity in our population. We have also found the existence of genetic interaction between *CAPN5 *and *PPARD *genes.

## Methods

### Population

The study sample comprised 1953 individuals, 725 obese (defined as BMI ≥ 30) and 1228 non obese subjects recruited from both a population based study and from outpatient visits to different hospitals in a multicenter study as previously described [35,36]. The referral centres involved in this research are Hospital Universitario de Valme (Sevilla), Hospital Universitario Ramon y Cajal (Madrid), and Hospital Universitario San Carlos (Madrid). Informed written consent was obtained from all study participants. The study protocol was designed in accordance with institutional guidelines for human research and was approved by the Ethics Committees of all referral centres.

### Polymorphisms

In order to perform genetic interaction analyses, we have selected three frequent and highly informative polymorphisms (heterozygosity ≥ 40%) within *CAPN5 *(rs7102149 G/A), *PPARG *(rs2938392 C/T) and *PPARD *(rs2076167 A/G, N798N). Since in our previous study the genetic association analysis of four *CAPN5 *polymorphisms with BMI was not conclusive, we have selected a polymorphism at the second intron of the gene, close to the recombination hot spot observed in this gene region that defines the two haplotypic blocks analysed in our prior report [30]. This polymorphism has been found to be expressed in two *CAPN*5 mRNAs where it is predicted to encode a tryptophan residue in the wild type state (G, T*G*G) and a stop codon in the polymorphic state (A, T*A*G). The *PPARG *polymorphism is located at the fifth intron of the gene in the same disequilibrium block than the Pro12Ala polymorphism using HapMap data, but have a higher allelic frequency and heterozygosity, being for these reasons more informative than Pro12Ala. The selected *PPARD *polymorphism encodes an asparagine (Asn) residue in the sixth exon of the gene.

### Genotyping

We obtained 5 ml of peripheral blood from all patients and controls to isolate germline DNA from leukocytes. DNA extraction was performed in a MagNa Pure LC Instrument (Roche Diagnostics, Indianapolis, IN, USA) according to the manufacturer's instructions. To perform polymerase chain reactions (PCRs), we prepared aliquots of DNA at a concentration of 5 ng/μl. The rest of the stock was cryopreserved at -20°C.

PCR reactions were run on a thermal cycler machine (MJ Research Inc., Waltman, Masachusetts, USA) using a final volume of 20 μl. The genotypes were carried out by using the pyrosequencing technology protocols [37]. The selected primers for pyrosequencing analysis are shown in Table S1. The pyrosequencing machine was programmed in accordance with the manufacturer's recommendation (Biotage, Uppsala, Sweden).

A 10% of the samples were re-extracted and retyped as an internal control of the quality of the genotyping with a concordance rate of > 99%.

### Statistical analysis

To analyze deviation from Hardy-Weinberg equilibrium, we used tests adapted from Sasieni [38] at the online resource available at the Institute for Human Genetics, Munich, Germany .

Logistic regression analysis was used to calculate individual and combined gene effects. For testing gene-gene interactions, we introduced and interaction term within the logistic regression model along with the univariate terms. All analyses have been adjusted for age, sex, smoking (defined as present or past history of smoking of at least five cigarettes per day for a minimum of 5 years), alcohol consumption (defined as a daily intake of more than 10 g) and physical activity. Power calculations suggest that we have 90% power at a significance level of 0.05 to detect an OR for obesity of 1.5.

### Statement of Ethics

We certify that all applicable institutional and governmental regulations concerning the ethical use of human volunteers were followed during this research.

## Results

### Monogenic association analysis

The observed allele frequencies were 0.63 in controls and 0.64 in cases at the *CAPN5 locus *(p = 0.456), 0.54 in controls and 0.53 in cases at the *PPARG locus *(p = 0.340) and 0.72 in controls and 0.69 in cases at the *PPARD locus*, showing the polymorphic allele at this last gene a slight over-representation in the obese subgroup (p = 0.021). All genotype frequencies are in accordance with the Hardy-Weinberg equilibrium law (p > 0.301) except for the cases group at the *PPARG *locus, with a slight deviation from the expected proportions (p = 0.037).

In the quantitative genotypic association analysis (Table 1), we didn't observe significant differences at *CAPN5 *or *PPARG loci*, whereas homozygotes for the polymorphic G allele at the *PPARD locus *have slightly greater BMI than individuals carrying at least one A allele (28.88 kg/m^2 ^vs 28.12 kg/m^2^, p = 0.033).

**Table 1:** Quantitative association analysis of the selected polymorphisms with BMI.

SNP	11	12	22	P (2df)
CAPN5 (rs7102149)	28,11 ± 1.01	28,14 ± 1.01	27,84 ± 1.01	0.552
	(0.820)	(0.592)	(0.281)	
PPARG (rs2938392)	28,27 ± 1.01	27,95 ± 1.01	28,21 ± 1.01	0.289
	(0.254)	(0.118)	(0.534)	
PPARD (rs2076167)	28,12 ± 1.01	28,15 ± 1.01	28,88 ± 1.01	0.103
	(0.409)	(0.697)	(**0.033**)	

Data are means ± std. error. Number in brackets are p values (1 df) resulting from the comparison of each genotype against the remaining genotypes. CAPN5, allele 1:G, allele 2: A; PPARG, allele 1: C, allele 2: T; PPARD, allele 1:A, allele 2: G

According to allelic frequencies and quantitative association analysis, the homozygous presence of the polymorphic G allele at *PPARD locus *is more common in the obese subgroup (OR = 1.43 [1.04–1.97], p = 0.027) (Table 2). No association was observed for the analysed polymorphisms at the *CAPN5 *(p ≥ 0.126) or *PPARG *(p ≥ 0.199) genes.

**Table 2:** Genetic association analysis of *CAPN5 *and *PPARD *gene variants with obesity.

Gene	Genotype	Controls	Cases	P value	OR [CI]
CAPN5 (rs7102149)	**GG**	498	299	0.899	1.01 [0.85–1.21]
	**GA**	550	332	0.342	1.09 [0.91–1.30]
	**AA**	180	94	0.126	0.82 [0.63–1.06]

PPARG (rs2938392)	**CC**	366	215	0.885	0.99 [0.81–1.20]
	**CT**	597	333	0.346	0.92 [0.77–1.01]
	**TT**	265	177	0.199	1.15 [0.93–1.43]

PPARD (rs2076167)	**AA**	639	348	0.295	0.91 [0.76–1.09]
	**AG**	494	300	0.843	0.98 [0.81–1.18]
	**GG**	95	77	**0.027**	1.43 [1.04–1.97]

### Interaction analysis

**Figure 1:**
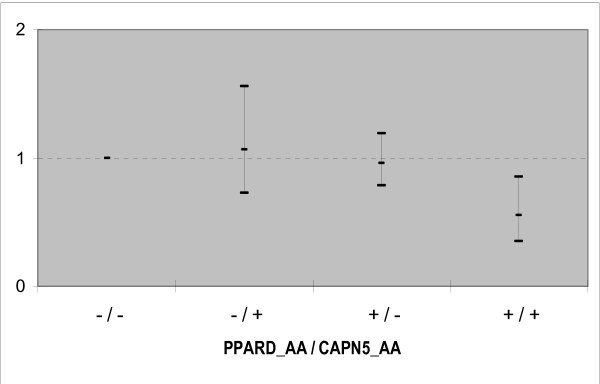
**Odds ratio (95% confidence interval) for the genetic interaction between the PPARD and CAPN5 genes in obesity.** Significant genetic interaction between the homozygous presence of the polymorphic allele at CAPN5 locus and the wild type at PPARD locus (CAPN5_AA*PPARD_AA), OR= 0.55; P value for  interaction = 0.038)

We didn't find evidence of interaction between *PPARD *and *PPARG *(p = 0.602), whereas a trend was observed for *PPARG *and *CAPN5 *genes (p = 0.056). Regarding *PPARD *and *CAPN5 *genes, we detected a significant genetic interaction between the homozygous presence of the polymorphic allele at *CAPN5 locus *and the wild type at *PPARD *locus (*CAPN5*_AA*PPARD_AA, p = 0.038) (Table 3 and figure 1). When compared with non carriers of any of the two genotypes, only the individuals carrying both genotypes are significantly less frequently obese (OR = 0.55 [0.35–0.85], p = 0.008).

**Table 3:** Odds ratio (OR) for obesity in the presence/absence of PPARD_AA and CAPN5_AA genotypes.

PPARD_AA	CAPN5_AA	Controls	Cases	OR [CI]	P value
-	**-**	502	317	1	
-	**+**	87	60	1.06 [0.72–1.56]	0.764
+	**-**	546	314	0.96 [0.78–1.19]	0.720
+	**+**	93	34	0.55 [0.35–0.85]	0.008

P value for interaction = 0.038

## Discussion

The PPARD receptor has a well established role in lipid metabolism, but its implication in the modulation of lipid stores is more controversial. Transgenic mice with targeted activation of *Ppard *in adipose tissue are resistant to both high-fat diet-induced and genetically predisposed (db/db) obesity; treatment of db/db mice with the PPARD agonist GW501516 also reversed obesity [11]. Several groups have examined different *PPARD *gene polymorphism in obesity related traits, half of them with positive results. All but two of these reports are limited to a SNP located at the 5'-UTR region of the exon 4 (rs2016520, referred as T+294C or c.-87T>C); this polymorphism is located at the linkage disequilibrium (LD) block adjacent to that of the N798N SNP analysed in the present work [16-19]. In the other two reports, tag SNPs throughout the genomic sequence of *PPARD *gene were explored, but only one of them obtained positive results [15,20]. The polymorphism analysed by our group has only been explored in the work by Grarup et al. [20], who didn't find association with obesity but identified an haplotype consisting of the rare alleles of this SNP and the rs2076169 polymorphism, located upstream in the same LD block, that was associated with a greater insulin sensitivity. We have not found evidence of the N798N polymorphism being associated with glucose levels or HOMA values in our population (data not shown). The identification of genetic association of *PPARD *with obesity in our population contributes to the notion that this gene is a candidate gene for obesity and related phenotypes.

We have not found association of the *PPARG *selected polymorphism with obesity in this population. We selected this polymorphism because is more informative than Pro12Ala and is in the same LD block. Only two works have previously analysed the Pro12Ala polymorphism in Spanish population [39,40]. In one of them, association with obesity was observed only in men (210 individuals) whereas in the other report, statistical significance was only achieved when physical activity was taken into account. Taken together,*PPARG *does not seem to be a major locus for obesity in our population. Regarding insulin sensitivity, we have not found association of this polymorphism with fasting insulin levels nor HOMA values. However, previous studies in Spanish population have associated the Pro12Ala polymorphism with estimates of insulin sensitivity [39,41], so we can no rule out *PPARG gene *as a genetic factor influencing insulin action in our population.

The association analysis at the *CAPN5 *did not contribute to elucidate if this gene has, *per se*, a role in obesity, since we did not found statistical significant differences at this *locus *between controls and cases (p ≥ 0.126). Thus, this polymorphism, which is predicted to encode a stop codon in one of the *CAPN5 *mRNAs identified so far, doesn't seem to be a functional variant involved in obesity.

The most relevant result of our work is the identification of genetic interaction between *CAPN5 *and *PPARD *genes. The physiological mechanism underlying the observed interaction is unknown, but we postulate that it could be related to mitochondrial biology. The existence of calpain activity in the mitochondria is a well known fact thanks to the extensive investigations performed in the field of apoptosis, one of the more relevant calpain biological activities. In this way, the CAPN5 homologue, CAPN10, has been shown to exist within the mitochondria and to be involved in pancreatic β-cell apoptosis, a process postulated to underlie the association of this gene with the insulin resistance phenotype [27,28,42]. On the other hand, mitochondrial versions of NRs have been identified, including the PPARG and the thyroid hormone receptor A (THRA) that is also a RXR partner in signalling, although the existence of the latter is still a controversial issue [43]. Moreover, some reports have described proteolytic activity of calpains over members of the PPAR and RXR families related to the generation of mitochondrial versions of these receptors that regulate mitochondrial gene expression [31]. Proteolytic cleavage of RXR by calpains has been also observed in hepatoma cell lines [32]. Recently, a report by Yajima et al. [33] describes that calpain system regulates the differentiation of adult primitive mesenchymal ST-13 adipocytes through the cleavage of PPARG. On the other hand, transcriptional regulation of calpain activity by PPARG has been also described [34]. In this way, a recent report has shown that the PPARG agonist rosiglitazone reduces μ-calpain activity, normalizing platelet endothelial cell adhesion molecule-1 levels and partially restoring platelet sensitivity to nitric oxide synthase inhibition in type 2 diabetic subjects, a mechanism related to the anti-atherogenic properties of this drug [44]. Although our results regarding CAPN5 and PPARG interaction are not conclusive, we can not rule out the possibility that exits given the statistical trend observed, the similitude between the PPAR family members and the existent bibliography. Further analysis in bigger populations or in other phenotypes besides functional studies will help to clarify this issue.

The existence of calpain activity over NRs, could exist not only inside the mitochondria, but also in the nucleus, being a possible biological explanation for the unusual presence of proteases into the nucleus; this cellular location has been observed for both CAPN5 and CAPN10 among other calpains [45]. Several authors have described calpain activity over diverse NRs including the androgen receptor [46,47] and the estrogen receptors (ERs) [48,49]. Interestingly, mitochondrial versions of the ERs have been also described [43]. Conversely, several NRs modulate the expression of calpains: retinoids, the ligands for retinoid acid receptors (RARs), estrogens, that mediates their action through the estrogen receptors (ERs) and the 1,25-dihydroxyvitamin D3, which uses the vitamin D receptor (VDR), have shown to regulate calpain activity [50-54].

Interaction genetic analysis can help to identify genetic factors that when acting together, modify the risk of suffering from a complex disease and contribute to elucidate the molecular mechanism of the studied disease. This knowledge could have direct implications in the clinical management of the patients, heading the pharmacological therapy to the most suitable biological process. We propose that the pathological mechanism of CAPN5 and, perhaps, its homologue CAPN10, could be related to the existence of a cross-talk between this calpain and the RXRs/PPARs nuclear receptors, which are key proteins in the intermediate metabolism and major pharmacological targets. For one hand, CAPN5 regulates these NRs levels and, on the other hand, the NRs regulate CAPN5 level through the induction/repression of its expression. However, functional studies are needed to investigate if the proposed mechanism could be plausible in living cells and if true, pharmacological implications for PPAR agonist should be explored.

## Competing interests

The authors declare that they have no competing interests.

## Authors' contributions

MES and RRL have genotyped samples, analysed and interpreted data and draft the manuscript, FJM, AG-P and AR have contributed to conception and design of the study and revised the manuscript, AG, LM MTML, JSH and MSR have contributed to the acquisition and interpretation of data and revised the manuscript.

## References

[B1] Bell CG, Walley AJ, Froguel P (2005). The genetics of human obesity. Nature reviews.

[B2] Rankinen T, Zuberi A, Chagnon YC, Weisnagel SJ, Argyropoulos G, Walts B, Perusse L, Bouchard C (2006). The human obesity gene map: the 2005 update. Obesity (Silver Spring, Md.

[B3] Korner A, Kiess W, Stumvoll M, Kovacs P (2008). Polygenic contribution to obesity: genome-wide strategies reveal new targets. Frontiers of hormone research.

[B4] Romao I, Roth J (2008). Genetic and environmental interactions in obesity and type 2 diabetes. Journal of the American Dietetic Association.

[B5] Gulick T, Cresci S, Caira T, Moore DD, Kelly DP (1994). The peroxisome proliferator-activated receptor regulates mitochondrial fatty acid oxidative enzyme gene expression. Proceedings of the National Academy of Sciences of the United States of America.

[B6] Tontonoz P, Hu E, Graves RA, Budavari AI, Spiegelman BM (1994). mPPAR gamma 2: tissue-specific regulator of an adipocyte enhancer. Genes & development.

[B7] He W, Barak Y, Hevener A, Olson P, Liao D, Le J, Nelson M, Ong E, Olefsky JM, Evans RM (2003). Adipose-specific peroxisome proliferator-activated receptor gamma knockout causes insulin resistance in fat and liver but not in muscle. Proceedings of the National Academy of Sciences of the United States of America.

[B8] Hevener AL, He W, Barak Y, Le J, Bandyopadhyay G, Olson P, Wilkes J, Evans RM, Olefsky J (2003). Muscle-specific Pparg deletion causes insulin resistance. Nature medicine.

[B9] Shulman AI, Mangelsdorf DJ (2005). Retinoid x receptor heterodimers in the metabolic syndrome. The New England journal of medicine.

[B10] Tonjes A, Scholz M, Loeffler M, Stumvoll M (2006). Association of Pro12Ala polymorphism in peroxisome proliferator-activated receptor gamma with Pre-diabetic phenotypes: meta-analysis of 57 studies on nondiabetic individuals. Diabetes care.

[B11] Wang YX, Lee CH, Tiep S, Yu RT, Ham J, Kang H, Evans RM (2003). Peroxisome-proliferator-activated receptor delta activates fat metabolism to prevent obesity. Cell.

[B12] Tanaka T, Yamamoto J, Iwasaki S, Asaba H, Hamura H, Ikeda Y, Watanabe M, Magoori K, Ioka RX, Tachibana K, Watanabe Y, Uchiyama Y, Sumi K, Iguchi H, Ito S, Doi T, Hamakubo T, Naito M, Auwerx J, Yanagisawa M, Kodama T, Sakai J (2003). Activation of peroxisome proliferator-activated receptor delta induces fatty acid beta-oxidation in skeletal muscle and attenuates metabolic syndrome. Proceedings of the National Academy of Sciences of the United States of America.

[B13] Leibowitz MD, Fievet C, Hennuyer N, Peinado-Onsurbe J, Duez H, Bergera J, Cullinan CA, Sparrow CP, Baffic J, Berger GD, Santini C, Marquis RW, Tolman RL, Smith RG, Moller DE, Auwerx J (2000). Activation of PPARdelta alters lipid metabolism in db/db mice. FEBS letters.

[B14] Oliver WR, Shenk JL, Snaith MR, Russell CS, Plunket KD, Bodkin NL, Lewis MC, Winegar DA, Sznaidman ML, Lambert MH, Xu HE, Sternbach DD, Kliewer SA, Hansen BC, Willson TM (2001). A selective peroxisome proliferator-activated receptor delta agonist promotes reverse cholesterol transport. Proceedings of the National Academy of Sciences of the United States of America.

[B15] Shin HD, Park BL, Kim LH, Jung HS, Cho YM, Moon MK, Park YJ, Lee HK, Park KS (2004). Genetic polymorphisms in peroxisome proliferator-activated receptor delta associated with obesity. Diabetes.

[B16] Aberle J, Hopfer I, Beil FU, Seedorf U (2006). Association of peroxisome proliferator-activated receptor delta +294T/C with body mass index and interaction with peroxisome proliferator-activated receptor alpha L162V. Int J Obes (Lond).

[B17] Aberle J, Hopfer I, Beil FU, Seedorf U (2006). Association of the T+294C polymorphism in PPAR delta with low HDL cholesterol and coronary heart disease risk in women. International journal of medical sciences.

[B18] Gouni-Berthold I, Giannakidou E, Faust M, Berthold HK, Krone W (2005). The peroxisome proliferator-activated receptor delta +294T/C polymorphism in relation to lipoprotein metabolism in patients with diabetes mellitus type 2 and in non-diabetic controls. Atherosclerosis.

[B19] Robitaille J, Gaudet D, Perusse L, Vohl MC (2007). Features of the metabolic syndrome are modulated by an interaction between the peroxisome proliferator-activated receptor-delta -87T>C polymorphism and dietary fat in French-Canadians. Int J Obes (Lond).

[B20] Grarup N, Albrechtsen A, Ek J, Borch-Johnsen K, Jorgensen T, Schmitz O, Hansen T, Pedersen O (2007). Variation in the peroxisome proliferator-activated receptor delta gene in relation to common metabolic traits in 7,495 middle-aged white people. Diabetologia.

[B21] Lagou V, Scott RA, Manios Y, Chen TL, Wang G, Grammatikaki E, Kortsalioudaki C, Liarigkovinos T, Moschonis G, Roma-Giannikou E, Pitsiladis YP (2008). Impact of peroxisome proliferator-activated receptors gamma and delta on adiposity in toddlers and preschoolers in the GENESIS Study. Obesity (Silver Spring, Md.

[B22] Hautala AJ, Leon AS, Skinner JS, Rao DC, Bouchard C, Rankinen T (2007). Peroxisome proliferator-activated receptor-delta polymorphisms are associated with physical performance and plasma lipids: the HERITAGE Family Study. American journal of physiology.

[B23] Stefan N, Thamer C, Staiger H, Machicao F, Machann J, Schick F, Venter C, Niess A, Laakso M, Fritsche A, Haring HU (2007). Genetic variations in PPARD and PPARGC1A determine mitochondrial function and change in aerobic physical fitness and insulin sensitivity during lifestyle intervention. The Journal of clinical endocrinology and metabolism.

[B24] Saez ME, Ramirez-Lorca R, Moron FJ, Ruiz A (2006). The therapeutic potential of the calpain family: new aspects. Drug Discov Today.

[B25] Gao G, Dou QP (2000). N-terminal cleavage of bax by calpain generates a potent proapoptotic 18-kDa fragment that promotes bcl-2-independent cytochrome C release and apoptotic cell death. Journal of cellular biochemistry.

[B26] Horikawa Y, Oda N, Cox NJ, Li X, Orho-Melander M, Hara M, Hinokio Y, Lindner TH, Mashima H, Schwarz PE, del Bosque-Plata L, Horikawa Y, Oda Y, Yoshiuchi I, Colilla S, Polonsky KS, Wei S, Concannon P, Iwasaki N, Schulze J, Baier LJ, Bogardus C, Groop L, Boerwinkle E, Hanis CL, Bell GI (2000). Genetic variation in the gene encoding calpain-10 is associated with type 2 diabetes mellitus. Nature genetics.

[B27] Johnson JD, Han Z, Otani K, Ye H, Zhang Y, Wu H, Horikawa Y, Misler S, Bell GI, Polonsky KS (2004). RyR2 and calpain-10 delineate a novel apoptosis pathway in pancreatic islets. The Journal of biological chemistry.

[B28] Arrington DD, Van Vleet TR, Schnellmann RG (2006). Calpain 10: a mitochondrial calpain and its role in calcium-induced mitochondrial dysfunction. Am J Physiol Cell Physiol.

[B29] Gonzalez A, Saez ME, Aragon MJ, Galan JJ, Vettori P, Molina L, Rubio C, Real LM, Ruiz A, Ramirez-Lorca R (2006). Specific haplotypes of the CALPAIN-5 gene are associated with polycystic ovary syndrome. Human reproduction (Oxford, England).

[B30] Saez ME, Martinez-Larrad MT, Ramirez-Lorca R, Gonzalez-Sanchez JL, Zabena C, Martinez-Calatrava MJ, Gonzalez A, Moron FJ, Ruiz A, Serrano-Rios M (2007). Calpain-5 gene variants are associated with diastolic blood pressure and cholesterol levels. BMC medical genetics.

[B31] Casas F, Daury L, Grandemange S, Busson M, Seyer P, Hatier R, Carazo A, Cabello G, Wrutniak-Cabello C (2003). Endocrine regulation of mitochondrial activity: involvement of truncated RXRalpha and c-Erb Aalpha1 proteins. Faseb J.

[B32] Matsushima-Nishiwaki R, Shidoji Y, Nishiwaki S, Moriwaki H, Muto Y (1996). Limited degradation of retinoid X receptor by calpain. Biochemical and biophysical research communications.

[B33] Yajima Y, Sato M, Sorimachi H, Inomata M, Maki M, Kawashima S (2006). Calpain system regulates the differentiation of adult primitive mesenchymal ST-13 adipocytes. Endocrinology.

[B34] Hodgkinson CP, Ye S (2003). Microarray analysis of peroxisome proliferator-activated receptor-gamma induced changes in gene expression in macrophages. Biochemical and biophysical research communications.

[B35] Martinez-Larrad MT, Fernandez-Perez C, Gonzalez-Sanchez JL, Lopez A, Fernandez-Alvarez J, Riviriego J, Serrano-Rios M (2005). [Prevalence of the metabolic syndrome (ATP-III criteria). Population-based study of rural and urban areas in the Spanish province of Segovia]. Medicina clinica.

[B36] Ramirez-Lorca R, Grilo A, Martinez-Larrad MT, Manzano L, Serrano-Hernando FJ, Moron FJ, Perez-Gonzalez V, Gonzalez-Sanchez JL, Fresneda J, Fernandez-Parrilla R, Monux G, Molero E, Sanchez E, Martinez-Calatrava MJ, Saban-Ruiz J, Ruiz A, Saez ME, Serrano-Rios M (2007). Sex and body mass index specific regulation of blood pressure by CYP19A1 gene variants. Hypertension.

[B37] Ronaghi M, Karamohamed S, Pettersson B, Uhlen M, Nyren P (1996). Real-time DNA sequencing using detection of pyrophosphate release. Analytical biochemistry.

[B38] Sasieni PD (1997). From genotypes to genes: doubling the sample size. Biometrics.

[B39] Gonzalez Sanchez JL, Serrano Rios M, Fernandez Perez C, Laakso M, Martinez Larrad MT (2002). Effect of the Pro12Ala polymorphism of the peroxisome proliferator-activated receptor gamma-2 gene on adiposity, insulin sensitivity and lipid profile in the Spanish population. European journal of endocrinology / European Federation of Endocrine Societies.

[B40] Ochoa MC, Marti A, Azcona C, Chueca M, Oyarzabal M, Pelach R, Patino A, Moreno-Aliaga MJ, Martinez-Gonzalez MA, Martinez JA (2004). Gene-gene interaction between PPAR gamma 2 and ADR beta 3 increases obesity risk in children and adolescents. Int J Obes Relat Metab Disord.

[B41] Soriguer F, Morcillo S, Cardona F, Rojo-Martinez G, de la Cruz Almaraz M, Ruiz de Adana Mde L, Olveira G, Tinahones F, Esteva I (2006). Pro12Ala polymorphism of the PPARG2 gene is associated with type 2 diabetes mellitus and peripheral insulin sensitivity in a population with a high intake of oleic acid. The Journal of nutrition.

[B42] Giguere CJ, Covington MD, Schnellmann RG (2007). Mitochondrial calpain 10 activity and expression in the kidney of multiple species. Biochemical and biophysical research communications.

[B43] Lee J, Sharma S, Kim J, Ferrante RJ, Ryu H (2007). Mitochondrial nuclear receptors and transcription factors: Who's minding the cell?. Journal of neuroscience research.

[B44] Randriamboavonjy V, Pistrosch F, Bolck B, Schwinger RH, Dixit M, Badenhoop K, Cohen RA, Busse R, Fleming I (2007). Platelet sarcoplasmic endoplasmic reticulum Ca2+-ATPase and {micro}-Calpain activity are altered in type 2 diabetes mellitus and restored by rosiglitazone. Circulation.

[B45] Gafni J, Hermel E, Young JE, Wellington CL, Hayden MR, Ellerby LM (2004). Inhibition of calpain cleavage of huntingtin reduces toxicity: accumulation of calpain/caspase fragments in the nucleus. The Journal of biological chemistry.

[B46] Libertini SJ, Tepper CG, Rodriguez V, Asmuth DM, Kung HJ, Mudryj M (2007). Evidence for calpain-mediated androgen receptor cleavage as a mechanism for androgen independence. Cancer research.

[B47] Pelley RP, Chinnakannu K, Murthy S, Strickland FM, Menon M, Dou QP, Barrack ER, Reddy GP (2006). Calmodulin-androgen receptor (AR) interaction: calcium-dependent, calpain-mediated breakdown of AR in LNCaP prostate cancer cells. Cancer research.

[B48] Murayama A, Fukai F, Murachi T (1984). Action of calpain on the basic estrogen receptor molecule of porcine uterus. Journal of biochemistry.

[B49] Shiba E, Kim S, Fujitani M, Kambayashi JI, Kawamura I, Tsujimoto S, Shimomura K, Tanji Y, Taguchi T, Kimoto Y, Izukura M, Takai SI (1996). Possible involvement of calpain in the growth of estrogen receptor positive breast cancer cells. Anticancer research.

[B50] Nishikiori N, Osanai M, Chiba H, Kojima T, Ohguro H, Sawada N (2007). Inhibitory effects of retinoic acid receptor alpha stimulants on murine cataractogenesis through suppression of deregulated calpains. Investigative ophthalmology & visual science.

[B51] Sribnick EA, Matzelle DD, Ray SK, Banik NL (2006). Estrogen treatment of spinal cord injury attenuates calpain activation and apoptosis. Journal of neuroscience research.

[B52] Gamerdinger M, Manthey D, Behl C (2006). Oestrogen receptor subtype-specific repression of calpain expression and calpain enzymatic activity in neuronal cells--implications for neuroprotection against Ca-mediated excitotoxicity. Journal of neurochemistry.

[B53] Aranceta J, Perez Rodrigo C, Serra Majem L, Ribas Barba L, Quiles Izquierdo J, Vioque J, Tur Mari J, Mataix Verdu J, Llopis Gonzalez J, Tojo R, Foz Sala M (2003). [Prevalence of obesity in Spain: results of the SEEDO 2000 study]. Medicina clinica.

[B54] Reichrath J, Rech M, Seifert M (2003). Vitamin D-induced apoptosis and melanoma: does calpain represent the major execution protease rather than caspases?. The Journal of pathology.

